# Padina Minor Extract Confers Resistance against Candida Albicans Infection: Evaluation in a Zebrafish Model

**DOI:** 10.3390/biology13060384

**Published:** 2024-05-27

**Authors:** Chang-Cheng Wu, De-Sing Ding, Yi-Hao Lo, Chieh-Yu Pan, Zhi-Hong Wen

**Affiliations:** 1 Department of Marine Biotechnology and Resources, National Sun Yat-Sen University, Kaohsiung 804, Taiwan; 2 Department of Obstetrics and Gynecology, Zuoying Armed Forces General Hospital, Kaohsiung 81342, Taiwan; 3 Department and Graduate Institute of Aquaculture, National Kaohsiung University of Science and Technology, Kaohsiung 811213, Taiwan; dsding@nkust.edu.tw; 4 Department of Family Medicine, Zuoying Armed Forces General Hospital, Kaohsiung 81342, Taiwan; loveangelsome@yahoo.com.tw; 5 Department of Nursing, Shu-Zen Junior of Medicine and Management, Kaohsiung 82144, Taiwan

**Keywords:** *Candida albicans*, treatment, growth, survival, *Padina minor*

## Abstract

**Simple Summary:**

This study is the first to evaluate the effect of feeding zebrafish with *Padina minor* extract on preventing and treating *Candida albicans* infections. This study showed that 1% *P. minor* extract effectively improved the resistance of zebrafish against *C. albicans* infection. The results of this study are expected to be useful for preventing and treating *C. albicans* infections in fish.

**Abstract:**

*Padina minor* is a seaweed rich in polysaccharides often used in food, feed, fertilizers, and antibacterial drugs. This study is the first to evaluate the effect of feeding zebrafish with *Padina minor* extract on preventing and treating *C. albicans* infections. This study evaluated the growth, survival, and disease resistance effects of *P. minor* extract on zebrafish. The fish were divided into four groups: three groups treated with 1%, 5%, or 10% *P. minor* extract and one untreated group (c, control). Subsequently, we analyzed how the extract affected the immune function of zebrafish infected with *C. albicans*. Based on the lethal concentration (LC50) calculated in the first stage, 1% was used as the effective therapeutic concentration. The results showed that the growth rate of the 1% feed group was the best, and no significant difference in survival rates between the four groups was observed. Feeding with 1% *P. minor* extract downregulated the expression of key inflammatory genes like tumor necrosis factor-alpha (TNF-α), interleukin-1β (IL-1β), and IL-10, effectively preventing and treating *C. albicans* infections in zebrafish. This study is a preliminary evaluation of the therapeutic efficacy of *P. minor* extracts against *C. albicans*.

## 1. Introduction

Aquatic organisms can be used to initially evaluate treatments for animal and human diseases, making cross-field contributions to aquatic and biomedical research. The zebrafish (*Danio rerio*) is a primitive vertebrate that has surpassed other animals and become the most used model organism because of its many advantages [1]. This study evaluated the therapeutic effect of *Padina minor* extract in zebrafish infected with *Candida albicans*.

Vulvovaginal candidiasis is a common form of human vaginitis caused by fungal infections, of which *C. albicans* is the most common pathogen. *C. albicans* is present in healthy vaginal environments. However, in a suitable environment, *C. albicans* proliferates in large quantities, affecting human health. Currently, the medical costs associated with the treatment of *C. albicans* infections are considerable [2]. Recent studies indicate that fungal diseases cost more than USD 7.2 billion, with inpatient care accounting for USD 4.5 billion and outpatient care accounting for USD 2.6 billion [3]. Research shows that at least 700,000 people die from antibiotic-resistant microbial infections annually, and *C. albicans* have developed drug-resistant strains [4]. Therefore, seeking safer and natural medicines is important.

Seaweeds are basic producers in the ocean and are rich in nutrients. Seaweeds can be used as a feed additive in aquaculture and for water quality management in aquaculture [5,6,7,8,9,10,11,12]. In addition, seaweeds are also used in biomedicine [13], fertilizer [10], and cosmetic products [14]. Seaweeds have important physiological activities, including antioxidant, hypolipidemic, antiviral, anti-tumor, and immune effects [15]. Algae cells are rich in nutrients and active substances, promoting animal health, advances in medicine, and health care value, which makes research on algae increasingly attractive worldwide [16]. Seaweed polysaccharides are one of the most widely used seaweed components and are mainly divided into alginate, fucoidan, agar, agarose, and carrageenan [17]. Different seaweed polysaccharides have distinct functions. For example, sodium alginate is used in food processing, immobilization matrices [18], dental impression materials [19], tissue engineering [20], and drug carriers [21,22]. Fucose and sulfate ester groups in fucoidans are widely used in health foods [23]. Moreover, seaweeds have several pharmacological properties, such as anticancer, antibacterial, antifungal, etc. [24,25]. Their extracts and purified ingredients can be used for their antiviral, anti-inflammatory, hypolipidemic, hypoglycemic, antimelanogenic, anti-bone-loss, hepatoprotective, and anticoagulant effects [26,27,28,29,30].

*Padina minor* is an important seaweed in the brown alga group which is native to and commonly found in Taiwan. *P. minor* is a common species in Taiwanese waters and is distributed throughout Japan, the Ryukyu Islands, South Korea, Taiwan, China, the Philippines, Thailand, and Guam. Fantail algae possess anti-inflammatory and antibacterial properties [31]. Fantail algae extract is rich in polysaccharides, such as sulfated polysaccharides [32], and is often used in food, feed, fertilizers, and antibacterial drugs. *P. minor* is an important aquaculture alga. If the natural extract of *P. minor* can improve the health of aquatic animals and reduce the use of synthetic drugs, it will be of great value for developing the aquaculture industry and reaching sustainable development goals (SDGs).

Zebrafish is an excellent model organism because its entire genome has been sequenced, and its embryos grow fast and are easy to observe. Zebrafish genes are 70% similar to human genes, making them important research subjects in toxicology, teratology, pathology, cancer, and other related fields. This study used zebrafish as an animal model for *C. albicans* infection and used *P. minor* extract to treat this fungal infection. In addition to treating fungal diseases in fish, this study also evaluates the efficacy of using *P. minor* extract as an alternative initial treatment for vaginitis infections.

## 2. Materials and Methods

### 2.1. Effects of Dietary P. minor on Growth, Survival, and Disease Resistance of Zebrafish

#### 2.1.1. Zebrafish

The zebrafish [*Danio rerio* (AB Line) sub-adults] used in this study were purchased from Gin Star Technology Co., Ltd. (New Taipei City, Taiwan). They were raised in the Breeding Department of the Kaohsiung University of Science and Technology in a 1-ton fiber-reinforced plastic (FRP) tank. The photoperiod during the breeding was 10 L:14 D. The animals were fed twice daily, and the feed amount was approximately 5% of the total body weight. The water quality parameters are shown in Table 1.

#### 2.1.2. Alga Extract

*P. minor* were obtained from an aquaculture farm in Taiwan. The algae were first freeze-dried and then crushed into algae powder. The ground and dried powder of *P. minor* algae was mixed with 1 mL^−1^ double-distilled water (ddH2O) per gram in a ratio of 1:5, warmed to 100 °C, extracted for 1 h, cooled, and then centrifuged at 6000× *g* for 30 min to obtain the extract. The supernatant was then mixed with the feed.

#### 2.1.3. Feed Preparation

Feed preparation was based on a commercially available feed formula. The feed ingredients are listed in Table 2. Each component remained unchanged except for the content of *P. minor* extract. Three experimental groups were fed with *P. minor* extract in concentrations of 1, 5, and 10%. The control group (C) was fed the formula without the addition of *P. minor* extract (n = 3). Three isoenergetic diets (18 MJ/kg gross energy (GE)) were formulated to provide crude protein (CP) levels from 34 to 35%; non-protein energy was about 16.0–16.7. Each feed group was first mixed evenly with a mixer, and then an appropriate amount of water was added to stir the mixture until the consistency was thick. Finally, a feed machine was used to squeeze the feed into strips, dry the feed with cold air at 20 °C, and then beat the feed to a suitable consistency using a homogenizer. Feed pellets were finally sealed into plastic bags and frozen at −20 °C for later use.

#### 2.1.4. Experimental Grouping

The zebrafish were first bred for one week to adapt and then screened for body size. Before starting the experiment, all zebrafish were tested for diseases to confirm that there were no pathogenic bacteria. Zebrafish of similar size and good health were selected and randomly placed in groups in a rectangular FRP tank (46 × 34 × 23 cm). The water level was 20 cm, and an air pump was used to increase the amount of dissolved oxygen. The fish were divided into four groups of ten fish each: 1%, 5%, and 10% *P. minor* extract and a control group (C). Feeding times were 8:00 am and 05:00 pm. After eight weeks, the survival rate, weight gain (WG), and feed efficiency (FE) of the zebrafish were recorded. Disease resistance experiments were conducted on fish infected with *C. albicans*.

Calculation formulas:

Survival rate (%) = Final fish count/Initial fish count × 100

Weight gain (WG) = (Final weight (g) − Initial weight (g)) [33].

Percentage of weight gain (WG (%)) = (Final body weight (g) − Initial body weight (g)/Initial body weight (g) × 100)

Feed efficiency (FE) = Final body weight (g) − Initial body weight (g)/Feed intake (g) [34].

#### 2.1.5. Disease Resistance Experiment

*C. albicans* used in this study was purchased from the Bioresource Collection and Research Center (BCRC, Hsinchu, Taiwan). Zebrafish with an average body length of 4 cm were used in this experiment. Each experiment was repeated three times; each group contained 30 fish (n = 30 fish). *C. albicans* were purchased from the Bioresource Collection and Research Center (Hsinchu, Taiwan). Before grouping the experiment, zebrafish were challenged with different concentrations of *C. albicans* to obtain the lethal concentration (LC50). Zebrafish fed with *P. minor* extract for two months were injected with 20 μL of *C. albicans* suspension (1 × 10^6^ CFU ml^−1^) into the caudal peduncle. The experiment lasted 168 h. Zebrafish death was monitored to evaluate the effective extract concentration.

### 2.2. Effects of Dietary P. minor Extract on Antibacterial and Immune Abilities of Zebrafish

#### 2.2.1. Immunity Test

Zebrafish infected with *C. albicans* were maintained in independent FRP tanks and continuously fed feed containing *P. minor* extract. Water temperature was maintained at 28 °C for seven days. The infection status of the zebrafish was observed daily, and the cumulative mortality rate was recorded. The LC50 was calculated and the value obtained was used for the immune function test using 1% *P. minor* extract. To evaluate the effects of *P. minor* extract on *C. albicans*, real-time quantitative polymerase chain reaction (RT-qPCR) was performed to analyze the changes in the fish immune system during *C. albicans* infection. Samples were collected after conducting in vivo disease resistance tests. The fish were collected at different times: 0, 6, 12, and 24 h after infection. After collection, the fish were immediately soaked in TRIzol reagent and stored on ice at −80 °C until RNA was extracted for subsequent testing.

#### 2.2.2. RNA Extraction

To extract total RNA, 30 zebrafish [*Danio rerio* (AB Line) sub-adults] were sacrificed at time points of 0 h (uninfected) and 6, 12, and 24 h after infection. Total RNA was extracted using the Nautia Gene^®^ NautiaZ Tissue Total RNA Mini Kit (Nautia Gene, Taipei, Taiwan). The extracted RNA was reverse-transcribed to cDNA. Then, the cDNA synthesized was placed in a RT-qPCR thermocycler using the SYBR Green System. The cDNA was then subjected to real-time PCR for β-actin, TNF-α, IL-1β, and IL-10 (GenBank accession no AF057040.1, AB183467.1, NM_212844.2, and AY887900.1). The total 10 μL PCR reaction mixture contained TOOLS 2X SYBR qPCR Mix (BIOTOOLS), 0.1 μM each of forward and reverse primers (Table 3), and 1 μL of sample cDNA, which was analyzed using an ABI PRISM 7500 Sequence Detection System (Applied Biosystems, Waltham, MA, USA). DEPC-MQ was used as a negative control (non-template control). The reaction program consisted of 50 °C for 2 min, 95 °C for 10 min, and 40 cycles of 95 °C for 15 s and 60 °C for 1 min, followed by a dissociation stage. The threshold cycle (Ct) of each gene was determined and normalized to β-actin mRNA levels. The experimental conditions and primer amplification efficiency were based on a previously published article by Pan et al., 2011 [35]. The data were then analyzed according to the relative gene expression calculated by 2^−ΔΔCt^ [36]. Refer to Table 3 to obtain the expression levels of the target genes with high accuracy. The primer sequences and gene names and functions listed are quoted from [35]. Cytokines related to the innate immune regulatory pathway triggered by LPS, including IL-1β, TNF-α, and IL-10, were analyzed in this study.

### 2.3. Statistical Analysis

Data were analyzed using GraphPad Prism 5. Statistical differences between groups were compared and analyzed using Student’s *t*-test. All data are presented as mean ± SDs. A *p*-value of <0.05 was considered significant.

## 3. Results

### 3.1. Effects of Dietary P. minor on Growth, Survival, and Disease Resistance of Zebrafish

#### 3.1.1. Fish Growth and Survival

**Figure 1:**
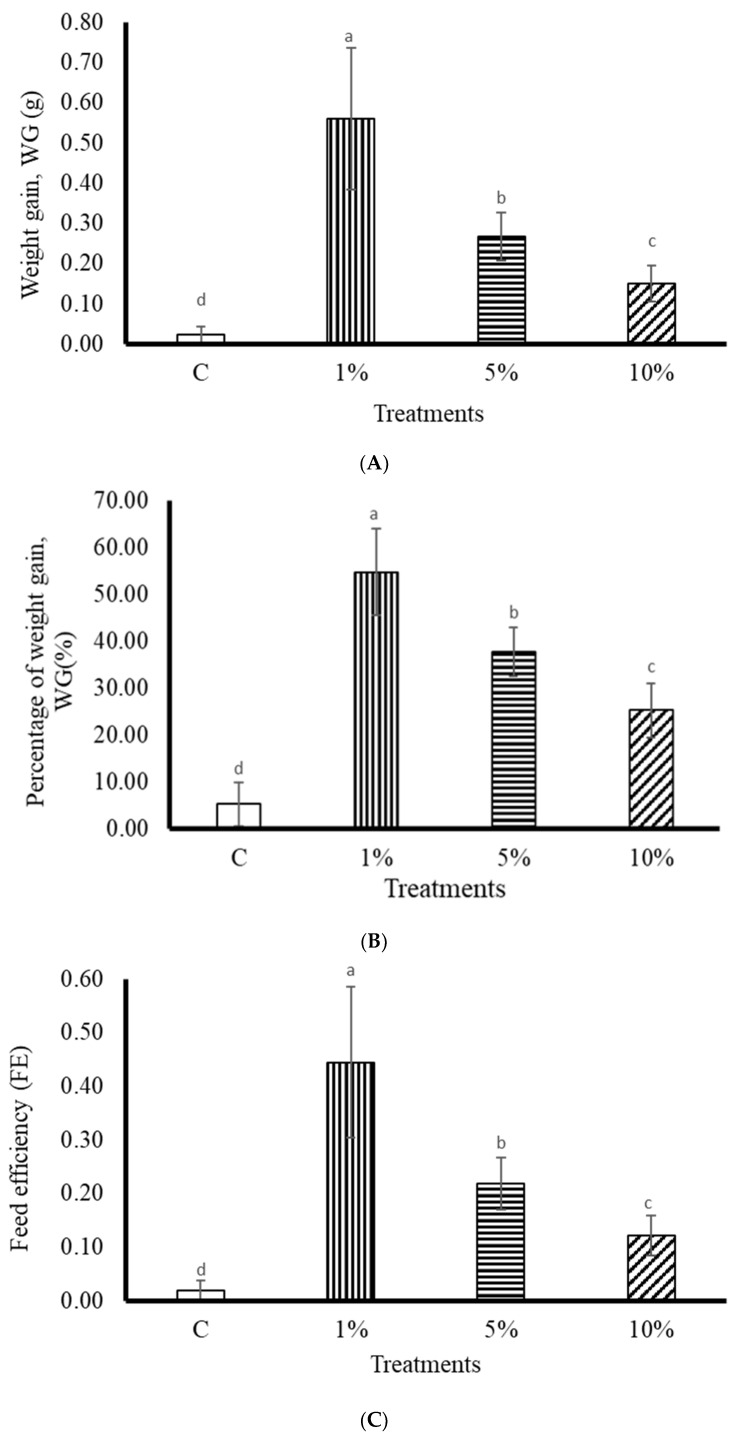
Effects of dietary *Padina minor* on (**A**) weight gain, (**B**) percentage of weight gain, and (**C**) feed efficiency of zebrafish. See calculations in Materials and Methods. Different letters indicate significant differences among groups (*p* < 0.05). Values are expressed as means ± SDs (n = 30 fish).

After eight weeks of feeding, the vitality and activity status of the zebrafish were normal. Significant changes in survival rate and body weight were observed. It was observed that fish fed with 1% extract had a better WG, with a significant difference compared to the other treatment groups (*p* < 0.05) (Figure 1A). As the feed concentration increased, WG decreased significantly (Figure 1B). When fish were fed 1% *P. minor* extract, FE reached 0.44 ± 0.14%, which was significantly different from the other treatment groups (Figure 1C). According to the experimental results, feeding with *P. minor* extract at a higher concentration led to stunted growth. The appropriate dose of *P. minor* extract was 1%; this resulted in improved growth. The survival rate results showed that no deaths occurred in any treatment group, and the survival rates were 100%.

#### 3.1.2. Disease Resistance Experiment

**Figure 2:**
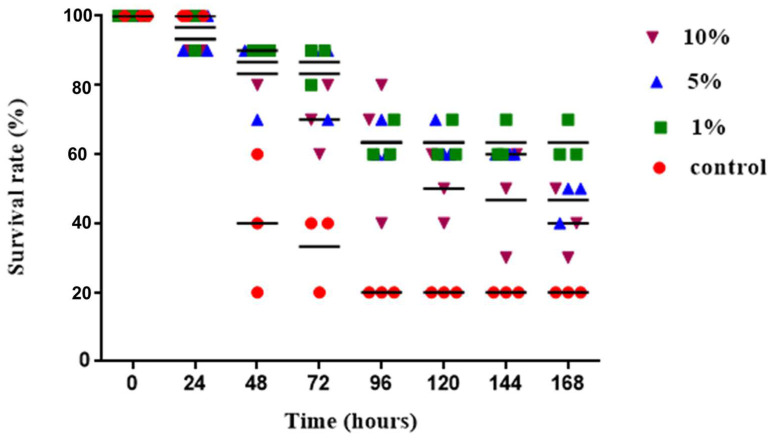
Changes in the survival rate of zebrafish fed with different concentrations of *P. minor* extract after *Candida albicans* infection. Each point in the graph represents the number of repetitions and fish. Values are expressed as means ± SDs (n = 30 fish).

Different concentrations of *P. minor* extract (1%, 5%, and 10%) were mixed with the feed, and after two months of feeding, a *C. albicans* suspension was injected. Mortality results showed that the survival rate of zebrafish in group C after 168 h was only 20%, whereas the survival rate of the 1% group was the highest, at 63%, followed by the 5% group (48%) and the 10% group (40%) (Figure 2). The experimental results showed that the 1% feed treatment group had a significantly better survival rate than the other treatment groups (*p* < 0.05).

### 3.2. Effects of Dietary P. minor Extract on Antibacterial and Immune Abilities of Zebrafish

#### Immunity

**Figure 3:**
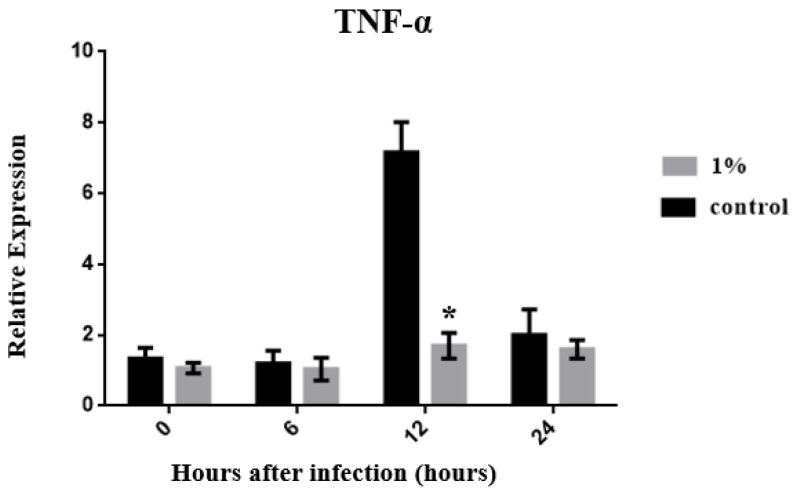
Effects of dietary *Padina minor* extract on TNF-α expression in zebrafish infected with *Candida albicans* at 0, 6, 12, and 24 h. Data are expressed as mean ± standard deviation (SD). * indicates significant difference between the control and treatment groups at different time points (*p* < 0.05, n = 15 fish).

**Figure 4:**
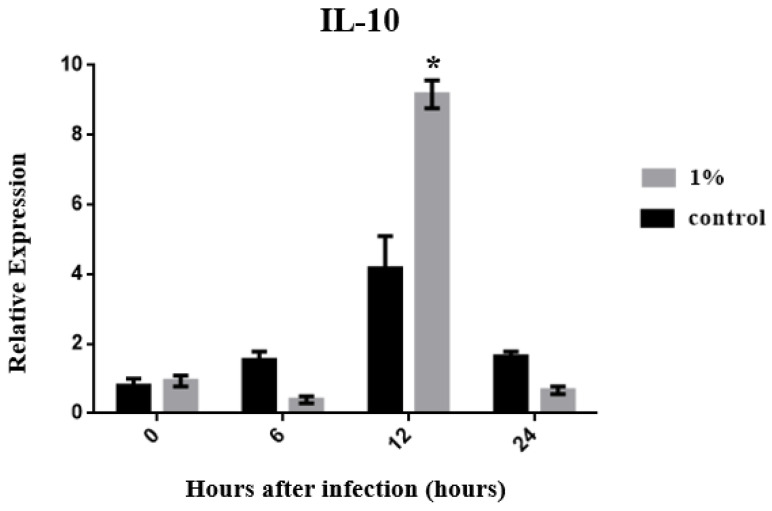
Effects of dietary *Padina minor* extract on IL-10 expression in zebrafish infected with *Candida albicans* at 0, 6, 12, and 24 h. Data are expressed as mean ± standard deviation (SD). * indicates significant differences between the control and treatment groups at different time points (*p* < 0.05, n = 15 fish).

**Figure 5:**
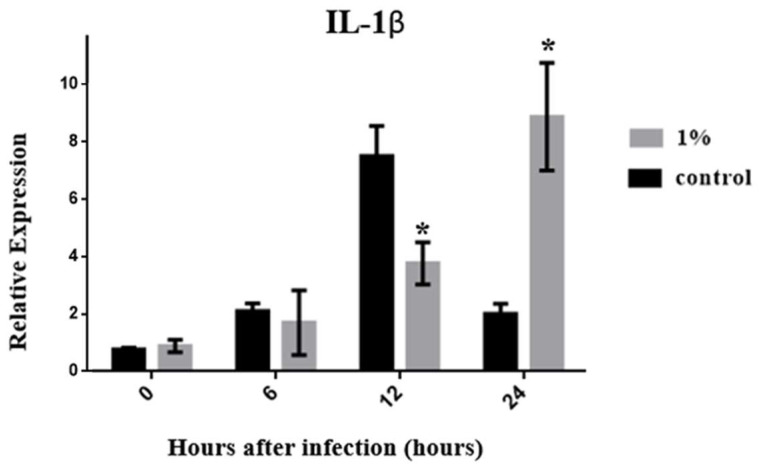
Effects of dietary *Padina minor* extract on IL-1β expression in zebrafish infected with *Candida albicans* at 0, 6, 12, and 24 h. Data are expressed as mean ± standard deviation (SD). * indicates significant differences between the control and treatment groups at different time points (*p* < 0.05, n = 15 fish).

According to the experimental results, the expression level of TNF-α in the control group reached a peak at 12 h, and a significant difference between the C and experimental groups was observed (*p* < 0.05) (Figure 3). The TNF-α expression level of the C group decreased over time, whereas its expression was stable in the experimental group. However, no significant difference in TNF-α expression was found at any time point compared with its expression level in the early stage of infection (*p* > 0.05). The expression of IL-10, an anti-inflammatory cytokine, reached its highest level at 12 h after infection in both groups. Only at this time point did the experimental group show a higher expression level of IL-10 than the C group (Figure 4). This may have occurred because TNF-α and IL-1β are promoted to produce an inflammatory response 12 h after infection, driving IL-10 to rapidly increase to combat the inflammatory response. As the inflammatory response weakens, the expression of IL-10 also gradually weakens after 24 h. The results of a cytokine involved in innate immune responses, IL-1β, showed a rapidly increasing expression level after 6 h in the C group, reached a maximum at 12 h, and then declined. However, the experimental group showed a delayed performance. The expression level of IL-1β began to increase only after 12 h, reaching a maximum at 24 h (Figure 5), demonstrating that zebrafish were beginning to mount a cellular immune response to fight infections. In summary, feeding 1% of *P. minor* extract changed the expression levels of TNF-α, IL-1β, and IL-10 in zebrafish, improving the immunity of the animal model. The exception was TNF-α, whose expression was reduced by *P. minor* extract.

## 4. Discussion

### 4.1. Fish Growth and Survival

The higher the concentration of *P. minor*, the more inhibited the growth of the zebrafish. The growth rate of the 1% treatment group was 28 times higher than that of the C group. When administered at an appropriate concentration, *P. minor* extract promotes zebrafish growth. Previous studies demonstrated a significant difference in body weight gain in rats fed high-fat diets with *Gracilariopsis lemaneiformis* extract compared to the control group [1]. Feeding with *Sargassum ilicifolium*, *Nizimuddinia zanardini*, *Cystoseira indica*, and *Padina australis* significantly affected the growth of *Litopenaeus vannamei* [37]. Therefore, food preference and nutrition may be the main reasons for the changes in the growth rate of zebrafish. Zebrafish eat benthic planktonic crustaceans, worms, and insect larvae [38]. In addition, they consume dipteran larvae that can effectively suppress mosquito populations [39]. In the present study, *P. minor* was added to the feed of zebrafish. Excessive doses of *P. minor* extract inhibit the growth of zebrafish; however, no deaths were observed during the experiment. Therefore, *P. minor* had a significant effect on the growth but not on the survival of zebrafish.

### 4.2. Application of Algae in Disease Resistance

Different concentrations of *P. minor* extract mixed with feed and fed for two months enhanced the immunity of zebrafish. Mortality rates were observed after injecting the pathogenic bacterial suspension. The results showed that the survival rate of group C after 168 h was only 20%, whereas the 1% group had the best survival rate (63%), followed by the 5% group (48%) and the 10% group (40%). In addition, the 1% treatment group had a significantly better survival rate than the other treatment groups. Therefore, feeding a high concentration of *P. minor* extract may weaken the antibacterial effect or affect bacterial digestion and metabolism, leading to death. Previous studies have shown that *P. minor* inhibits the growth of *Vibrio harveyi* [40] and that *P. australis* has a significant antibacterial activity against *Aeromonas hydropilla* [41]. The results of this study also showed that feeding *P. minor* could effectively inhibit the threat posed by *C. albicans* to zebrafish.

### 4.3. Immunity and Disease Resistance

According to previous research, in *Salmo gairdneri* Richardson, red blood cells bind to *C. albicans* after the infection; however, they do not kill *C. albicans* immediately. However, the presence of head kidney macrophages reduces the ability of red blood cells to bind to *C. albicans* and form rosettes [42]. Therefore, the fish immune system inhibits *C. albicans* infection. According to our results, the gene expression of TNF-α in the C group reached the highest level at 12 h post-infection, which was significantly different from that in each treatment group, and then gradually decreased. In contrast, the TNF-α gene expression in the treatment groups was stable, which may be because the *P. minor* extract induces the immune system of zebrafish and improves the inhibitory effect against *C. albicans* or the *P. minor* extract improves the anti-stress response ability of zebrafish. Previous studies have shown that the addition of *C. albicans* and *Pseudomonas aeruginosa* to the breeding environment of zebrafish leads to fungal epithelial invasion, swimbladder edema, and epithelial extrusion events, affecting the epithelium and respiratory organs of zebrafish [43]. However, these symptoms were not observed in any of the treatment groups in this study. We found that the respiratory rate of zebrafish infected with *C. albicans* was accelerated, which may be related to the stress response or disease infection.

In mammals, IL-4 and IL-13 synergize with IL-10 and are critical for balancing the immune response against pathogens and suppressing inflammation. IL-10 has a more significant anti-inflammatory effect than IL-4 and is critical for gill homeostasis [44]. In the present study, IL-10 gene expression reached its highest levels at 12 h post-infection, especially in the experimental group. This may have occurred because TNF-α and IL-1β are promoted to produce an inflammatory response 12 h after infection, driving IL-10 to rapidly increase to combat the inflammatory response. As the inflammatory response weakens, the expression of IL-10 also gradually weakens after 24 h. As the number of bacteria in the organism increases, the expression of IL-10 increases, and tissue repair and protection occurs over time, leading to a decrease in IL-10 expression. IL-10 is involved in various physiological processes in animals. However, IL-10 is mainly used to stimulate two immune responses, characterized by mucus and immunoglobulin E (IgE) production, eosinophilia, and macrophages. Many aspects of the immune response enhance and protect barrier surfaces, including mucus proliferation, tissue repair, and IgE blockade by toxins [45,46,47]. IL-10 is present in various types of fish [48,49]. IL-10 has been found in the kidneys, intestines, and gills of zebrafish, indicating that it plays a role in maintaining the homeostasis of these tissues. Therefore, the increase and decrease in IL-10 levels observed in this study may have been due to the in vivo regulatory effects of infection in zebrafish. However, according to the experimental results, IL-10 levels decreased significantly 24 h after treatment, indicating that the zebrafish were in the process of tissue recovery. The immune-related manifestations caused by the TNF-α, IL-1β, and IL-10 genes in zebrafish have been clearly described in the research report of Pan et al., 2011 [35].

Previous studies have demonstrated that when fish are infected with *Vibrio anguillarum*, IL-1β can be detected in the head kidney, blood, spleen, liver, gills, and peritoneum. IL-1β has a better macrophage monolayer expression ability [50]. In gilthead seabream, infection with *V. anguillarum* led to an increase in IL-1β gene expression [50]. Infection of *Larimichthys crocea* with *V. alginolyticus* also led to an increase in IL-1β gene expression [51]. Therefore, the expression level of IL-1β can be used as a basis for evaluating the degree of infection and the ability of the body to produce immunity. According to our results, the performance of the control group increased rapidly after 6 h and began to decline after 12 h. Feeding with 1% *P. minor* extract increased the performance only after 12 h, reaching the highest point at 24 h. According to the experimental results, it was observed that infected zebrafish began to produce a cellular immune response to slow down the expression of IL-1β.

In summary, 1% dietary *P. minor* extract reduces the expression of TNF-α, IL-1β, and IL-10 genes in zebrafish infected with *C. albicans*. The expression level of the IL-10 gene was significantly up-regulated at 12 h, and the expression level of IL-1β was significantly up-regulated at 24 h.

## 5. Conclusions

This study showed that 1% *P. minor* extract effectively improved the resistance of zebrafish against *C. albicans* infection. This is the first time that zebrafish have been used as a model organism for vaginal pathogenic fungal infection. The results of this study are expected to be useful for preventing and treating *C. albicans* infections in mammals and humans.

## Figures and Tables

**Table 1:** Zebrafish culture water quality conditions.

Water Quality	Numerical Value	Water Quality	Numerical Value
Temperature (°C)	28.00 ± 0.5	Dissolved oxygen (ppm)	5.00 ± 0.05
pH	7.3 ± 0.5	Ammonia nitrogen (ppm)	0.01 ± 0.05
**Nitrite** (ppm)	0.02 ± 0.05	Nitrate (ppm)	0.03 ± 0.05

**Table 2:** Algae additive feed formula for zebrafish.

	Treatment			
Ingredients (g)	Control	1%	5%	10%
Fishmeal	42	42	42	42
Soy flour	20	20	20	20
Squid powder	5	5	5	5
Cellulose	6.1	6	5.6	5.1
Starch	16.6	16.6	16.6	16.6
Fish oil	3.3	3.3	3.3	3.3
Shrimp powder	3	3	3	3
Mineral mix ^a^	2	2	2	2
Vitamin mix ^b^	2	2	2	2
*Padina minor* extract	0	0.1	0.5	1
Feed basic ingredients				
Crude protein	34.7	34.3	35.0	34.0
Fat	14	14	14	14
Ash content	11.8	11.8	11.8	13.7
Moisture	0.5	0.5	0.5	0.5

Mineral mix ^a^: Polyvita soluble, Hung chang pharmaceutical co., Ltd., Tainan, Taiwan, per kg: vitamin A 5,000,000 IU, vitamin D 1,000,000 IU, vitamin B1 2000 mg, vitamin B2 3000 mg, vitamin B6 1500 mg, vitamin B12 8000 μg, vitamin C 10,000 mg, vitamin E 1000 mg, vitamin k3 1000 mg, nicotinamide 10,000 mg, and folic acid 300 mg. Vitamin mix ^b^: cobalt 150 mg, copper 1200 mg, iodine 325 mg, iron 1500 mg, magnesium 6000 mg, manganese 1500 mg, potassium 100 mg, selenium 10 mg, sodium 5.9 mg, sulfur 0.72%, zinc 9600 mg, calcium 25.5%, and phosphorus 12.75%.

**Table 3:** The sequence of primers used in this study.

Number	Gene Name	Primer Sequence (5′→3′)
1	β-actinf (z)	AGAGCTATGAGCTGCCTGACG
2	β-actinr (z)	CCGCAAGATTCCATACCCA
3	TNF-α (z)	TGACTGAGGAACAAGTGCTTATGAG
4	TNF-α (z)	GCAGCGCCGAGGTAAATAGTG
5	IL-1β (z)	ATGGCAGAAGTACCTAAGCTC
6	IL- 1β (z)	TTAGGAAGACACAAATTGCATGGTGAACTCAGT
7	IL-10 (z)	CGCTTCTTCTTTGCGACTGTGCT
8	IL-10 (z)	TCACCATATCCCGCTTGAGTTCC

## Data Availability

The data presented in this study are available on request from the corresponding author.
